# The influence of tooth shade on near-infrared light transmission through human dentine and enamel: an in-vitro study

**DOI:** 10.1007/s10103-025-04304-2

**Published:** 2025-01-27

**Authors:** Sachin Kulkarni, Laurence J. Walsh, Monte James McEntyre, Roy George

**Affiliations:** 1 School of Medicine and Dentistry, Griffith University, Gold Coast, QLD Australia; 2 School of Dentistry, University of Queensland, Brisbane, QLD Australia; 3 School of Dentistry, University of Adelaide, Adelaide, SA Australia

**Keywords:** Photobiomodulation, Infrared, Shade, Wavelength, Substrate

## Abstract

This in-vitro study assessed the influence of the shade of human teeth on the transmission of near-infrared light. A total of 40 teeth were used. After cleaning the root surface and removing cementum, the teeth were sectioned into slices 3 mm thick, with each comprising a portion of the crown (enamel-dentine (ED)) and of the root (dentine only). The shade of the crown and the root was measured using a digital spectrophotometer. All samples were irradiated using 660, 808, or 904 nm diode lasers, and a multi-wavelength LED light source (700–1100 nm, Nuralyte^®^). Using a laser power meter, the percent transmission was calculated. Differences between Vita shade groups A, B, and C were analysed using ANOVA and post-hoc tests. Overall, dentine samples showed approximately 40% greater transmission than samples of enamel and dentine. There were significant influences for shade group and for sample thickness on the transmission of 660 nm light (*P* < 0.01), but not for other light sources. There was a statistically significant influence of light source on transmission. Across both crown and root samples, the ranking for light transmission from greatest to least was LED (700–1100 nm) > (904 nm = 808 nm) > 660 nm. Within the range from 660 to 1100 nm, the longer wavelengths are transmitted better by both enamel and dentine. The transmission of visible red light (660 nm) was affected by Vita tooth shade, while the transmission of near infrared light (700–1100 nm) was not affected by Vita shade.

## Introduction

Photobiomodulation (PBM) is a process where the interaction of light with specific types of molecules - photoacceptors or photoreceptors - produces a therapeutic biologic alteration. The source of such photons can be lasers or light-emitting diodes (LEDs) [1]. In dentistry, PBM has been applied for the reduction of inflammation and pain, with common wavelengths being 660 nm, 808 nm, and 904 nm [2–4]. These wavelengths align with major absorption peaks in cytochrome C oxidase and other photo-acceptors or photo-receptors that are involved in PBM.

When using PBM, a tooth, like all biological materials, is considered turbid, with both scattering and absorption events occurring. When a tooth is treated using PBM, some light is lost by scattering in the enamel and dentine, however it is essential that sufficient light reaches the dental pulp to activate the chromophores in dental pulp cells [5, 6]. There are several factors that influence the scattering and absorption of light within teeth [7]. Enamel factors include enamel thickness, translucency, opacity, shade, degree of maturation, and surface characteristics (such as roughness), while dentine factors include thickness, translucency, opacity, shade, sclerosis, the presence of smear layer, and the length and diameter of tubules [5, 6].

In a clinical setting, not all such enamel and dentine factors can be measured rapidly, except for tooth shade. One in vitro study reported reduced transmission of near infrared light over time as teeth darkened progressively following exposure to a tetracycline endodontic medicament (Ledermix™, Lederle Pharmaceuticals, Wolfrathausen, Germany) under conditions that were known to cause darkening of tooth structure [8]. However, there are no studies that describe the effect of natural shade variations in teeth on the transmission of near infrared light.

Visual methods for assessing shade are subjective, even among experienced clinicians, with only 26% inter-observer agreement reported [9–11]. To address this issue, a range of electronic shade measuring devices are available, including colorimeters and spectrophotometers. Spectrophotometers are not affected by object metamerism, and have better reproducibility and a longer working life than colorimeters [12]. The reported accuracy of the commonly used VITA Easyshade^®^ spectrophotometer is between 96 and 99% ^13^.

Based on these considerations, the aim of the present laboratory study was to assess the influence of natural variation of shade in human teeth on the transmission of light wavelengths used for PBM.

## Materials and methods

### Teeth preparation:

Teeth used in this study were extracted for orthodontic or periodontal reasons, with the approval of the Griffith University Human Research Ethics committee (Ref 2022/668). The inclusion criteria were that teeth were intact and had complete root development (a closed apex). Teeth were excluded if they had intense external staining that could not be removed, or if they had visible fractures, root resorption, surface defects, caries, restorations, or root canal fillings, when examined using a stereomicroscope (Olympus SZ51, Evident, Tokyo, Japan) at 10 X magnification. In addition, any teeth with shades that fell outside the normal range (where a shade could not be reported using a spectrophotometer) were also not included in the study.

A total of 40 single rooted extracted human teeth were collected. Cementum on the root surface was removed with an ultrasonic scaler (EMS^®^ Piezon, Switzerland) and a hand scaler (Sickle-hoe EverEdge^®^, Hu-Friedy, United States). The teeth were sectioned labio-lingually with a thin diamond disc (Superflex D915DF/22.0, Stoddard, Hertfordshire, UK). The thickness of the sections was checked using dental callipers (Iwanson ^®^, USA) set to 3 mm. Any of the samples that were damaged during sectioning (13 out of 80) were discarded at this point. To remove smear layer from the cut surface, sections were immersed in a bath of 18% (w/v) ethylenediaminetetraacetic acid (EDTA root canal irrigating solution, Ultradent, USA) for 2 min. This step was included because the presence of smear layer can attenuate near infrared light transmission through dentine (e.g. by scattering), and 2-minute treatment with EDTA can remove that smear layer [8, 13].

All samples were then washed with distilled water for 30 s to remove EDTA, then blotted to remove excess water, and stored in a moist state, in a light proof box to prevent any light-induced changes to tooth shade, until the samples were used to assess light transmission. The samples were kept moist to resemble the normal clinical situation of teeth in the mouth [14]. Light transmission was assessed separately for the crown region (containing enamel and dentine (ED)), and for the coronal third of the root (dentine only (D)).

### Shade measurement:

The lighting in the room at the benchtop level was measured using a professional digital light meter (Digitech, VIC, Australia) at 10,000 lx. This is the same level that would be expected in a clinical operatory when taking a shade clinically using a spectrophotometer, with the dental operating light turned off [15]. The spectrophotometer used to measure the shade of samples was the VITA Easyshade^®^ Compact (VE) (VITA Zahnfabrik H. Rauter GmbH & Co., Bad Säckingen, Germany). The VE device has a reported accuracy of 92.6%, and reproducibility of 96.4% ^13^. The measuring tip was applied at the same spot that was used to assess light transmission, namely the middle third of the crown sample and the coronal third of the root region. These regions were chosen because they are somewhat flat, and are representative of the locations where light would be delivered clinically to teeth. Before all sets of measurements, the automatic calibration cycle of the VE unit was completed. Single tooth measurement mode was selected, and the VITAPAN classical A1-D4 shade results recorded. This shade system was chosen as it is the most widely used system in clinical practice [16]. Under this shade system, A1 - A4 are reddish-brownish, B1 - B4 are reddish-yellowish, C1 - C4 are greyish shades, and D2 - D4 are reddish-grey [16]. Based on their shade, the 67 samples used in the study were placed into relevant groups (Table 1).

**Table 1:** Sample distribution according to shade group

HUE		A	B	C	Total
	CHROMA				
Enamel-Dentine	1	10	9	7	
	2	19	1	6	
	3	6	1	5	
	4	0	0	3	
	**Total**	35	11	21	**67**
Dentine	NA*	16	26	25	**67**

*Chroma was not used to assess dentine samples

### Light sources:

**Fig. 1:**
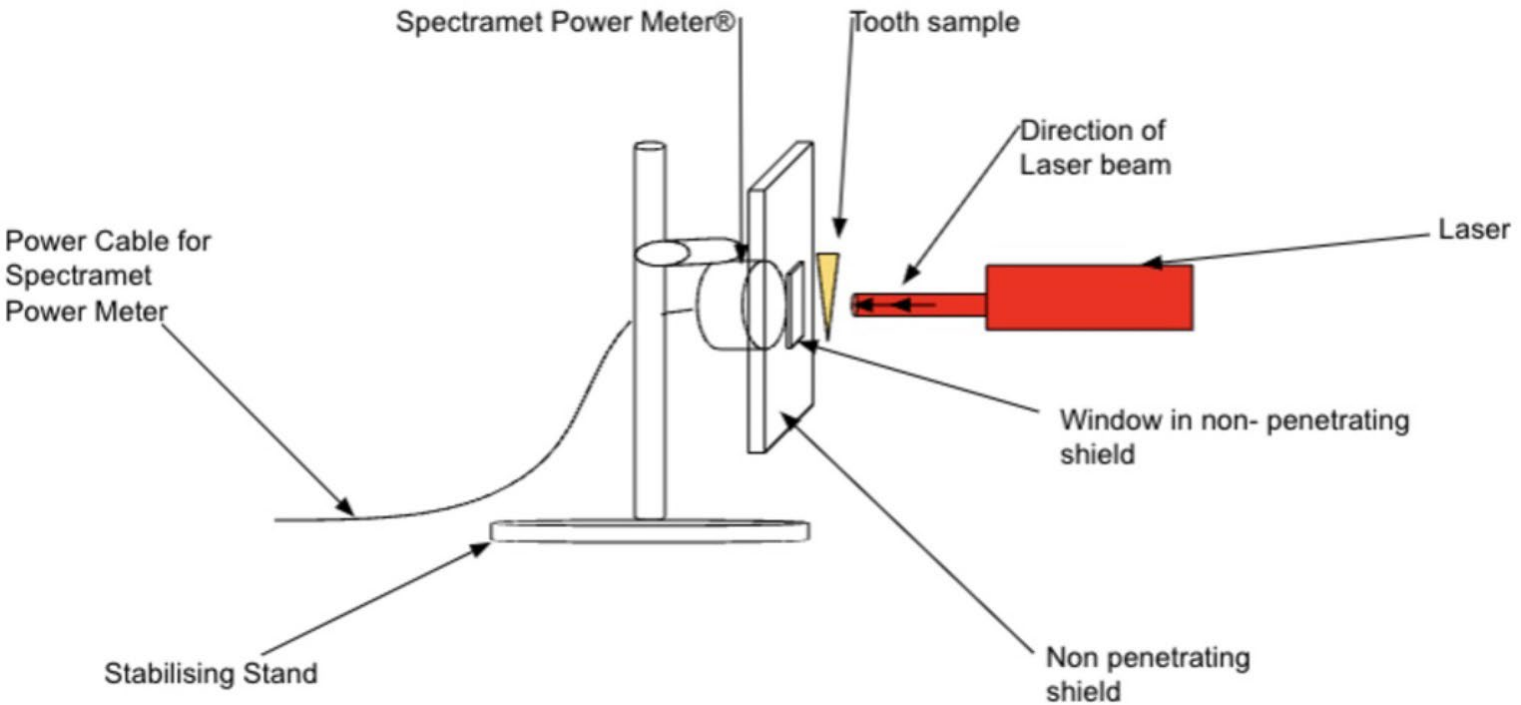
Experimental set up to measure light transmission

Details of the light sources used in this study are given in Table 2. All light sources used a fibre-optic guide similar to a dental curing light, with a similar spot size. The actual emitted light energy for all sources was measured using a laser power meter (SpectraMet, Laserdyne Technologies, QLD, Australia). This measurement was used as a baseline against which to compare the energy passing through the sample region of an opaque jig (Fig. 1). A 30 × 30 mm opaque jig was used, with a 5 × 5 mm window cut at its centre, behind which the sensor of the laser power meter was located. The window size of 5 × 5 mm was chosen as tooth samples were 6–10 mm in width, thus all of the window was covered by the crown or the root during light exposure runs.

**Table 2:** Light sources used in the study

Parameters	Konf^®^ Klas-D61	Konf^®^ Klas-D81	Irradia^®^	Nuralyte^®^ LED
Wavelength(s)	660 nm	808 nm	904 nm	700–1100 nm
Power	100 mW	110 mW	90 mW	110 mW
Power meter output reading	81.8 mW	100.1 mW	32.9 mW	113.0 mW
Measured spot size and calculated area	6 mm 0.28 cm^2^	6 mm 0.28 cm^2^	6 mm 0.28 cm^2^	7 mm 0.39 cm^2^
Power density	289 mW/cm^2^	354 mW/cm^2^	116 mW/cm^2^	293 mW/cm^2^
Total energy (for 60 s)	4.91 J	6.01 J	1.97 J	6.77 J
Energy density (for 60 s)	17.4 J/cm^2^	21.3 J/cm^2^	7.0 J/cm^2^	17.6 J/cm^2^

The workflow for each sample region was as follows. Once the sample was centred in the window, it was held in place with removable adhesive (Blu-Tak^®^, Bostik, Arkema, France). The distal end of the fibre-optic guide was then placed perpendicular to the tooth sample, in contact mode. The light source was activated for 60 s, and the reading monitored to ensure that the power meter reading was stable. The same irradiation time was used for all sample locations and for all light sources. The irradiation procedure was repeated 3 times, and the average reading used. This approach ensured that the results were reproducible. The same procedure was followed for both crown samples and root samples. The perpendicular orientation was used for four reasons. First, it is the most reproducible. Second, it gives the least loss by scatter from the sample surface. Third, it ensures the optimum transmission of light, even when the substrate is anisotropic, which is the case for tooth structure because of the prisms of enamel and the tubules of dentine, as well as the apatite crystals in both [17]. Finally, the perpendicular orientation is most relevant to clinical practice. Contact mode was used it is a reproducible position for mounted light tips, which is essential when comparing different light sources. Clinically, both contact and non-contact approaches (working distances from 2 to 4 mm) have been used for PBM, with similar results [18], even when the handpieces are handheld without any mounts for stabilisation.

### Analysis:

All statistical analyses used Instat^®^ software version 3.1 (GraphPad, San Diego, CA, USA) with *p* < 0.05 as the threshold for significance. The percent transmission was calculated for each of the two regions in each of the 67 samples for each light source, using the mean value from 3 replicate measurements. Group data were assessed for normality using the Kolmogorov-Smirnov test.

The statistical workflow was as follows. First, data on sample thickness were compared between VITA shade groups for both the crown and the root regions. This was to ensure that sample thickness was comparable between the shade groups. Second, to assess the influence of VITA shade on light transmission, data for each of the four light sources and each of the two locations (crown or root) were treated as a separate experiment. Within each of the resulting 8 experiments, the influence of VITA shade group on light transmission was assessed, using non-paired ANOVA. Where data sets did not follow a normal distribution, a non-parametric ANOVA based on ranks (the Kruskal-Wallis test) was conducted. Where a significant finding was made, an analysis of the relationship between sample thickness and light transmission was then made using least-squares linear regression. Third, for a given light source, transmission through the crown region of samples (enamel-dentine) and the root region (dentine only) was compared using repeated measures T tests. Finally, to assess the effect of light source, light transmission data for different light sources for the same samples were compared using repeated measures ANOVA and Tukey post-hoc tests, or Kruskal-Wallis and Dunn post-hoc tests, as appropriate. Group (enamel-dentine versus dentine) and sub-group analyses (shade sets A, B and C) were conducted between the four light sources.

## Results

A total of 67 samples were useable after sectioning. Once assigned to groups according to their shade, there were no significant differences in sample groups according to their thickness. There were no significant differences in thickness between crown and root samples.

### Influence of VITA Shade:

**Fig. 2:**
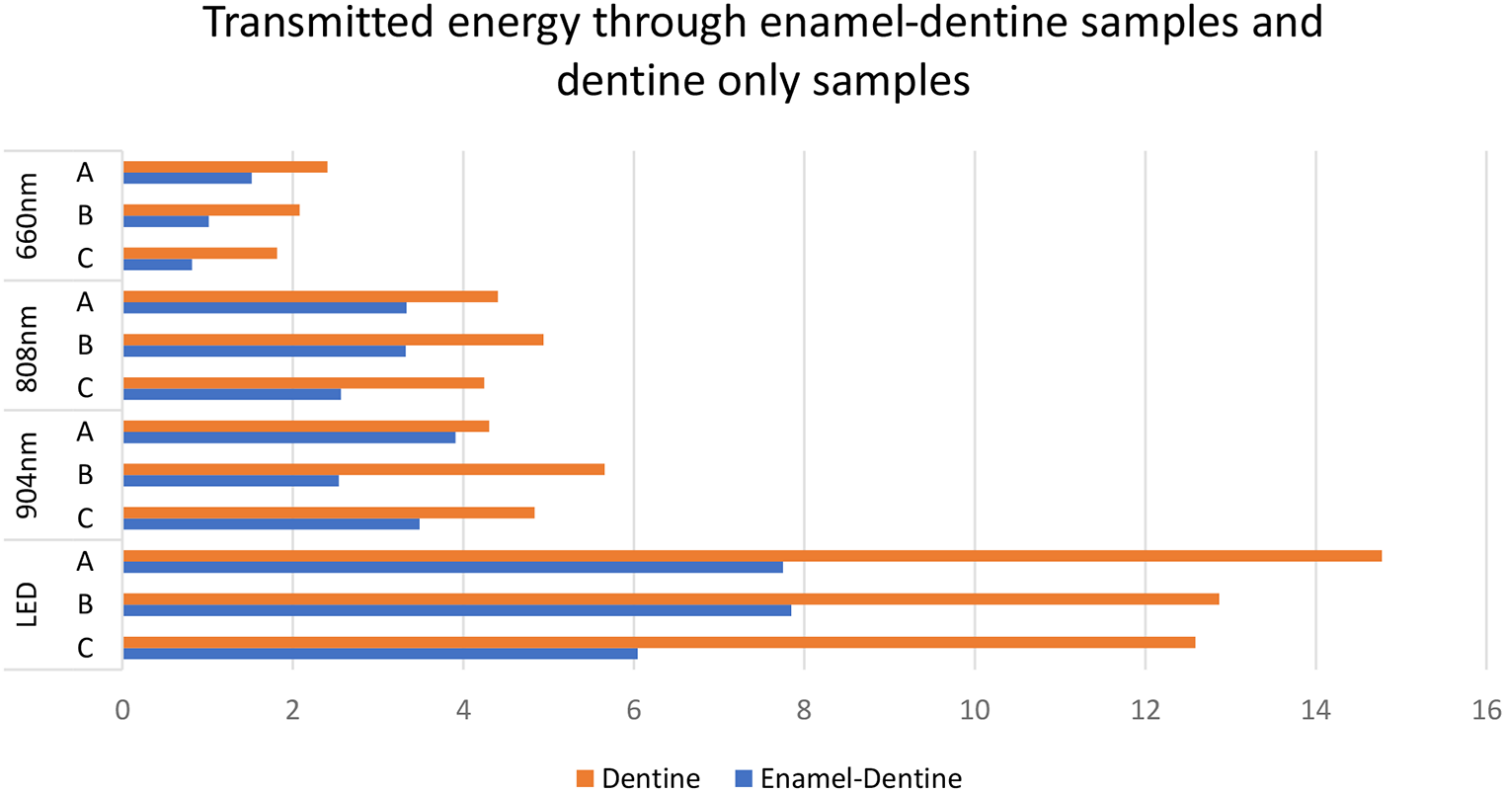
Percent light transmission for different light sources across the major shade groups (A, B and C). The consistent trends are that light transmission is better for dentine than for enamel-dentine; and that light transmission improves from 660 nm to 808 nm to 904 nm to the LED

Using each of the four light sources as an independent experiment, there was a statistically significant effect of shade on transmission for 660 nm light, between shades A and C for enamel-dentine crown samples (*p* < 0.01). No other significant differences were found (Fig. 2).

### Substrate thickness:

**Fig. 3:**
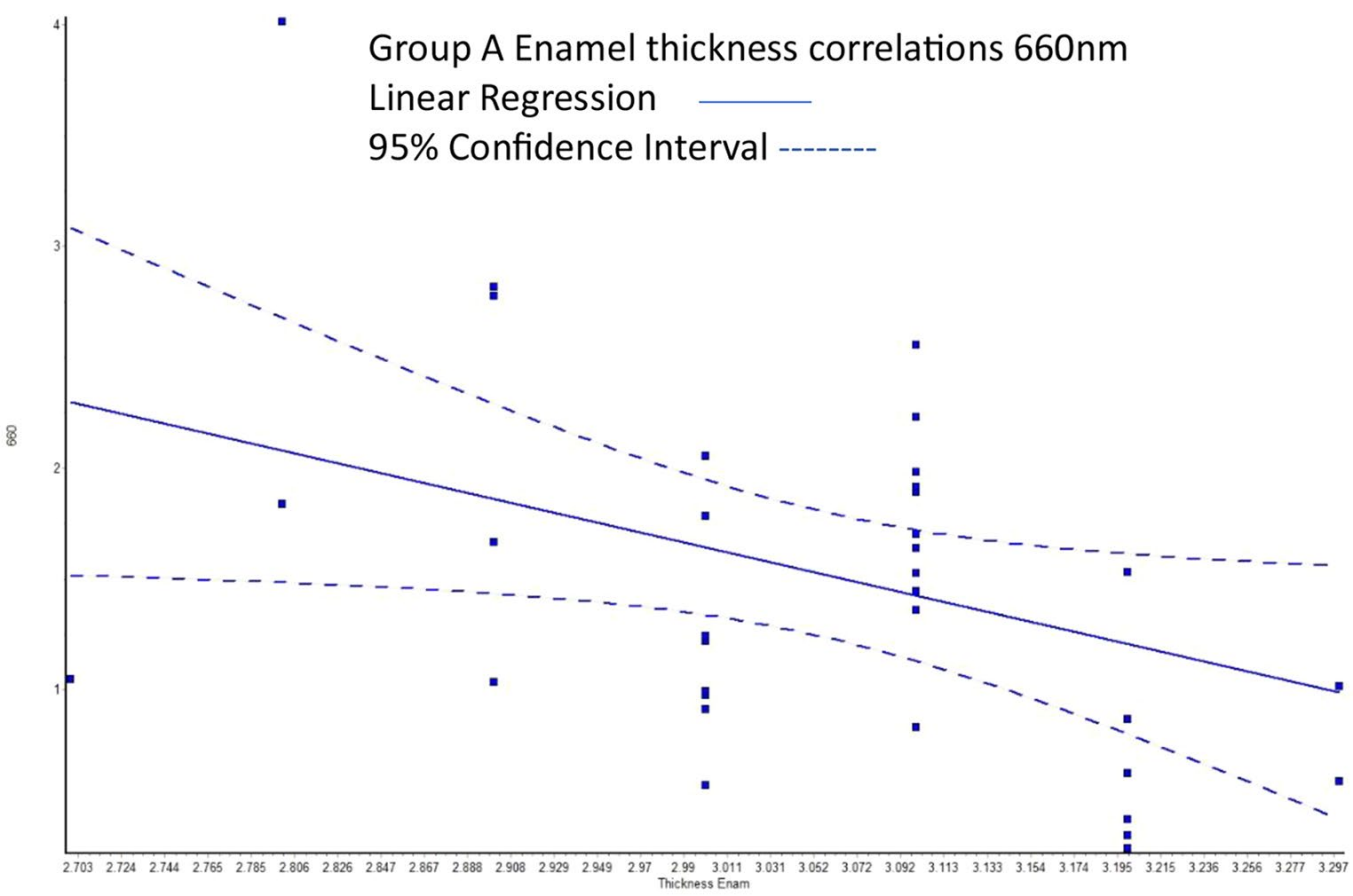
Linear regression model for A shades for 660 nm, showing that transmission reduced as enamel-dentine sample thickness increased

In enamel-dentine crown samples, as the thickness increased, the transmission of 660 nm light reduced (i.e. there was a negative linear correlation) (Fig. 3). A similar but weaker overall trend was seen for other diode laser light sources but in all cases it failed to reach the threshold for statistical significance.

### Substrate:

Differences in light transmission between enamel-dentine versus dentine only regions of the same samples were consistent. Taken as an average across all light sources, there was approximately 40% greater transmission in dentine. Transmission was significantly higher for dentine than for enamel-dentine across the four light sources and for all three shade groups (*P* < 0.05), except shade A with 904 nm, and shades A and B with 808 nm, which showed the same trend but failed to reach the threshold for statistical significance (Fig. 2).

### Light source:

**Fig. 4:**
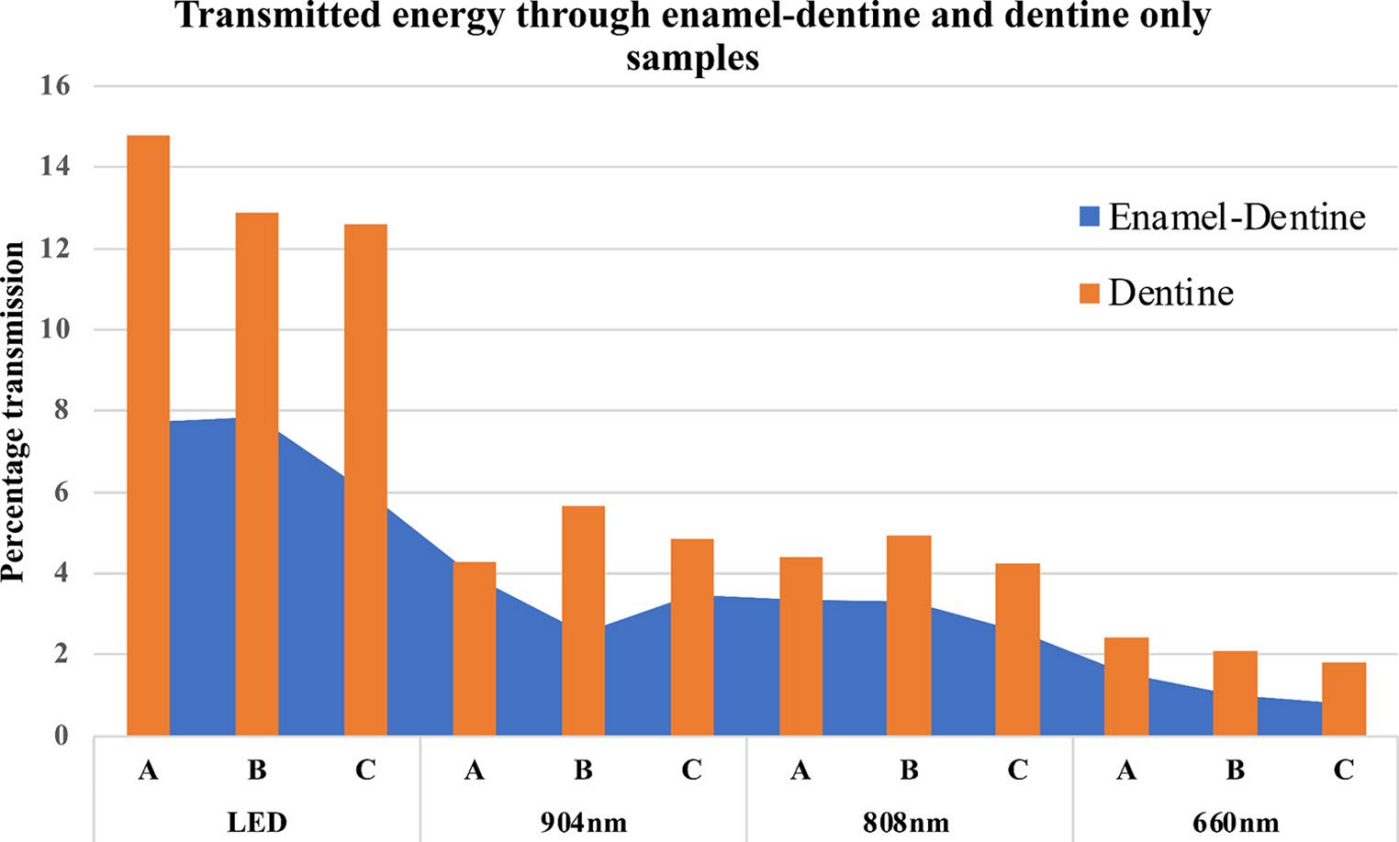
PBM Energy Transmission Percentages for Different Shade Groups as a Function of Wavelength

When matched for sample type (enamel-dentine or dentine) and shade group (A, B or C), light transmission was greater for the LED device than for any of the diode lasers (*p* < 0.05 in all cases), with the single exception of shade group C for enamel-dentine samples. There were no significant differences between 904 nm and 808 nm, but both were superior to 660 nm in all cases. Thus, the overall ranking from greatest to least for light transmission was LED > (904 nm = 808 nm) > 660 nm (see Fig. 4).

## Discussion

The present results reinforce the concept that near infrared light can transmit through human teeth to reach dental pulp, with consistently least transmission being that for 660 nm. Considering the importance of light penetration into teeth to reach the dental pulp for achieving PBM, the present results show that for visible red laser light (660 nm), tooth shade and thickness have significant effects. Moreover, the transmission of 660 nm light was found to be consistently less than for 808 nm, 904 nm, or for the LED device (700–1100 nm). These results infer that PBM treatments of the dental pulp with 660 nm light will be strongly influenced by shade and thickness, and that such factors should be considered carefully when choosing irradiation protocols.

On the other hand, the current results show that longer wavelengths in the near infrared are transmitted better, from 808 nm to 904 nm to 1100 nm. As this happens, with these longer there is less influence of shade and tooth thickness on light transmission. Clinically, this would mean that minor changes in the thickness of enamel and dentine that occur with age (e.g. due to abrasion or erosion) may not warrant changing laser parameters. Likewise, with the LED light source, there was no effect of shade or thickness, and the light source showed the strongest transmission of those tested, especially for root samples of dentine. This knowledge could simplify the use of light sources for PBM, as well as improve the efficacy of dental treatments involving light-based therapies for PBM.

The shade of teeth arises from the microstructural characteristics of enamel and dentine, especially how these influence the absorption, reflection and scatter of light [19, 20]. Both Mie and Rayleigh scattering occur within enamel, due to the presence of prisms and mineral crystals within those prisms. In contrast, dentine has a tubular microstructure, and tubules could act like waveguides for infrared light [19]. The present results reinforce this notion, with the finding that dentine transmits near infrared light much better than enamel and dentine.

A further consideration for tooth shade is that the present study used teeth without restorations and did not include teeth that have undergone endodontic treatments. The presence of various restorative and endodontic materials can cause internal staining of teeth, which could then reduce the transmission of light, as shown in past work [8]. As well as darkened tooth structure, there is also the possibility that dental materials themselves could absorb light, and that interfaces created by dental treatments (such as smear layers) could alter light transmission by causing greater scattering [8]. This is why the smear later from tooth sectioning was removed using EDTA in the present study. Further work is needed to assess how near infrared light transmission could be affected by dental treatments and their consequences.

Numerous publications have described the importance of wavelength on absorption in biological tissues [21, 22]. The present results add to these findings by showing the greater transmission of longer wavelengths in the progression from 660 nm to 1100 nm. One explanation for this may be that with increasing wavelength there is less Rayleigh scattering [19]. The LED device used had a wide wavelength range (700–1100 nm), and there is interest in using multiple wavelength devices for PBM with the aim of achieving greater PBM actions, based on the concept of activation of multiple chromophores [23]. It is already known that the penetration of light into soft tissues is enhanced by using multiple wavelengths in the range from 800 to 1000 nm [24], and the present results suggest that there a similar optical window for teeth.

It is important to consider that in the present study, each light source was delivered at 90 degrees to the sample surface in contact mode. This will place the orientation of the light source almost parallel to the dentinal tubules, which minimises the loss of energy as the light is directed to the dental pulp [17].This should help guide the light toward the pulp, despite some scatter events occurring along the journey [25].

None of the samples in the present study had dental caries. When pre-cavitation lesions of dental caries are present, an increase in light scattering may occur due to the presence of water and more surface porosity, while carious lesions in dentine may show reduced scattering of light [26, 27]. Porphyrins in dentine dental caries may absorb laser emissions, and reduce the light reaching the dental pulp [26]. Additionally, the present study did not consider structural changes in teeth due to age or traumatic injuries, such as dentine sclerosis, that also can influence the transmission of light [17, 28, 29]. There is the further caveat that the present study did not use intact teeth but rather sectioned teeth, which raises the possibility that the procedure of sectioning the tooth into halves could itself alter the optical properties of the tooth [17, 28–30]. Hence, some caution is needed when applying the present results to the clinical setting.

The configuration used in the present study directed the light towards the pulp chamber from buccal and lingual surfaces at the middle third of the crown, which corresponds to the position of the pulp horns [17, 28, 29], and involves the light being aimed at 90 degrees to the dentine-enamel junction [27]. The location and angulation of the light delivery tip will affect which areas of the pulp receive the highest dose of light. Further work is needed to explore how changing the position and angulation of the light delivery tip changes the effectiveness of PBM when treating an individual tooth.

Other limitations of this study include that not every possible tooth shade was included, with shades outside the range of the shade guide being excluded. Future studies could assess teeth with unusually light or dark shades outside the normal range, as well as teeth with unusual shades. Future work could also consider deciduous rather than permanent teeth, and include teeth that have been treated with restorative materials or that have undergone endodontic treatments. The present study did not include any teeth with cracks or other defects. In fact, care was taken to prevent damage during preparation of samples, and damage was checked for with magnification. In real-world clinical practice, transmission of visible red and near infrared light could be affected by fracture lines [31–33], as well as by other factors that affect the dentine, including dentine sclerosis due to age, traumatic injuries, or the response of the dentine to dental caries once cavitation has occurred. While the present study used a constant thickness of sample, it was not possible to control the composition of the dentine. This is an important limitation of the present work.

Further studies should also assess the impact of tooth shape and type as well as thickness. This would be relevant to dosage differences for small teeth (e.g. mandibular incisors) versus large teeth (e.g. maxillary first molars). Such studies would provide information to inform clinical protocols for PBM using diode laser or LED devices.

The results of the present study reinforce the concept that near infrared light can pass through tooth structure to reach the dental pulp. Previous work has shown that visible red light at 660–670 nm does reach the dental pulp, and can cause biostimulation of dental pulp cells when applied at 2–4 J/cm^2^, as shown in histological studies of extracted teeth [33, 34]. Likewise, near infrared light is transmitted well by dentine [17, 28]. It is important to consider that obstacles to light transmission will impair effective delivery of photons to target tissues. Absorption of light in tooth structure could result in photothermal changes, which are not desirable for the dental pulp. Warming the teeth is not the intended purpose when PBM is being undertaken, instead the effect should be achieved without heat or any unpleasant sensations being experienced by the patient. Based on the present results, one would predict the least heating with the light source that is transmitted the best, in this case the LED source. A recent clinical trial showed that the same LED light source used in this study when applied to healthy premolar teeth for 60 s was effective for PBM-induced analgesia, but did not cause discomfort in any subject. Under comparable exposure conditions for average power (100 mW) and energy density (17.6 J/cm^2^), in the same subjects and the same teeth on different days, diode lasers caused occasional discomfort (904 nm > 808 nm > 660 nm) [35]. This was attributed to photothermal changes. The association between light wavelengths and both desirable and undesirable effects should be explored further in clinical studies.

while the 904 and 808 nm lasers caused some sensations that subjects noticed.

In clinical practice, the adoption of photobiomodulation (PBM) devices by clinicians is influenced not only by their efficacy but also by factors such as cost, return on investment, training needs, radiation safety compliance, and integration into existing workflows. The findings indicate that further investigation into the dental applications of LED PBM devices is warranted, particularly due to their high transmission through dental structures, which may allow for reduced dosages and shorter treatment durations. This could enhance the value of PBM treatments, particularly for analgesia. Future clinical trials are necessary to compare different PBM devices, utilizing consistent exposure parameters for multi-wavelength LEDs and single-wavelength light sources.

## Conclusions

The results from this laboratory study show that visible red light (660 nm) is transmitted less well through tooth structure than longer wavelengths (808 to 1100 nm), and that dentine is better for light transmission than enamel. Tooth shade, when within the normal range, can be an important factor for the transmission of 660 nm, but not for near infrared light sources. The transmission of near infrared light (700–1100 nm) is not affected significantly by tooth shade. The present result for the ranking for light transmission also suggest that the LED light source was transmitted better than 808 and 904 nm lasers, which could have implications for PBM treatment protocols involving the dental pulp.
